# Palliative care reduces emergency room visits and total hospital days among patients with metastatic HPB and GI cancers

**DOI:** 10.1038/s41598-022-23928-w

**Published:** 2022-12-06

**Authors:** Angelle A. Billiot, Denise M. Danos, Jenny Stevens, Katie M. Vance, Mary C. Raven, John M. Lyons

**Affiliations:** 1 LSUHSC Department of Surgery, New Orleans, LA USA; 2 LSUHSC School of Public Health, New Orleans, LA USA; 3 Our Lady of the Lake-Division of Academic Affairs, Baton Rouge, LA USA; 4 Our Lady of the Lake Cancer Institute, 7777 Hennessy Blvd, Baton Rouge, LA 70808 USA

**Keywords:** Cancer, Outcomes research

## Abstract

Palliative care services (PCS) have improved quality of life for patients across various cancer subtypes. Minimal data exists regarding PCSfor metastatic hepatopancreaticobiliary (HPB) and gastrointestinal (GI) cancers. We assessed the impact of PCS on emergency department visits, hospital admissions, and survival among these patients. Patients with metastatic HPB and GI cancer referred to outpatient PCS between 2014 and 2018 at a single institution were included. We compared the demographics, outcomes, and end-of-life indicators between those who did and did not receive PCS. The study included 183 patients, with 118 (64.5%) having received PCS. There were no significant differences in age, gender, race, marital status, or insurance. Those receiving PCS were more likely to have colorectal cancer (*p* = 0.0082) and receive chemotherapy (*p* = 0.0098). On multivariate analysis, PCS was associated with fewer ED visits (*p* = 0.0319), hospital admissions (*p* = 0.0002), and total inpatient hospital days (*p* < 0.0001) per 30 days of life. Overall survival was greater among patients receiving PCS (HR: 0.65 (0.46–0.92)). Outpatient PCS for patients with metastatic HPB and GI cancer is associated with fewer emergency department visits, hospital admissions, and inpatient hospital days, and improved overall survival.

## Introduction

Detailed accounts of “appalling” conditions and “inadequate” symptom control for end-of-life patients began to surface in Europe in the 1950s and 60s^1–3^. A series of invited lectures given by British physician, Cicely Saunders, to doctors and nurses at Yale University in the early 1960s drew attention to these problems in the United States (US). Greater attention was given to these disparities in 1969 when Elisabeth Kubler-Ross published the groundbreaking book On Death and Dying^4^. Kubler-Ross argued for the patient’s inherent right to independently determine his own end-of-life care, while outlining a novel concept of dignity and peace during the dying process. These events engendered a national movement and eventually led to the formation of the first US hospice in Branford, Connecticut in 1973^5^.

In the subsequent 10 years, hospice care became recognized as a critical piece of the patient care continuum. The eventual funding of hospice care occurred as an amendment to the Social Security Act of 1982. As end-of-life care matured, the American Board of Internal Medicine recognized the need for a specialty training in palliative care medicine and formalized this as a board certification in 2008. By 2010, two-thirds of hospitals in the US offered palliative care services (PCS)^6^Several reports have demonstrated that PCS improves physical symptom control and enhances psychological health inadvanced cancer patients^7^. A landmark randomized controlled trial in 2010 showed that PCS not only improves quality of life and mood in non-small cell lung cancer patients, but it also improves median survival by two months despite less aggressive care^8^. Others have associated PCS in cancer patients with decreased use of chemotherapy, fewer hospitalizations near the end-of-life, and lower cost of care to both patients and healthcare systems^9–11^. Additionally, adverse effects associated with palliative care are minimal^12^.

However, most studies have analyzed the effects of PCS among groups of patients with many different types of cancer, and it is possible that the benefits of palliative care may be amplified in certain subtypes of symptomatic cancers while potentially less significant in others. At our institution, we have previously reported on the benefits of PCS among patients with head and neck cancer—a malignancy notorious for debilitating airway issues, secretion control, and a host of other challenging symptoms^13^. However, very little has been written regarding the role of PCS among patients with advanced gastrointestinal (GI) or hepaticopancreaticobiliary (HPB) cancer. This study was undertaken in an attempt to determine an association between PCS and end-of-life metrics in patients with terminal GI or HPB malignancies.

## Methods

The outpatient Palliative and Supportive Care Clinic is a free-standing space where patients are evaluated by a board-certified palliative care physician (MCR) and/or a palliative care nurse practitioner at each visit. Unless additional time is needed, initial clinic visits typically last an hour while follow up visits are 30 min each. Patients are generally seen on a monthly basis unless more frequent visits are warranted. All new patients are given the Patient Health Questionnaire-9 (Pfizer, Inc)—to screen for depression, anxiety, and somatoform disorders—and the Opioid Risk Tool to assess the risk of aberrant opioid use^14^.The content of the clinic visit depends upon the patient’s needs; however, the primary focus includes enhancement of illness understanding, pain/symptom management, advance care planning, caregiver education, and transition to hospice when appropriate. Referrals to the Palliative and Supportive Care Clinic are made entirely at the discretion of the treating provider.

The study protocol was reviewed by the Louisiana State University Health Sciences Center – New Orleans Institutional Review Board (LSUHSC-NO IRB; IRB protocol #19-1396) and was determined to be exempt under 45CFR46.104(d), Category #4.iii. A waiver of HIPAA was granted by the LSUHSC-NO IRB due to the terminal status of patients in a retrospective study. All methods in this study were carried out in accordance to relevant guidelines and regulations, including the Declaration of Helsinki.

We identified all patients who were referred to the outpatient PCS clinic from 2014 to 2018. We included only patients who were at least 18 years old and were referred to PCS for end-of-life care regarding an incurable malignancy from a GI or HPB primary source. End of life care refers to care given to patients who have stopped or who are in the process stopping treatment to control their disease. We excluded patients who were initially seen by the PCS team as an inpatient. Additional exclusion criteria were harboring a second malignancy which was neither GI nor HPB, receiving treatment at an outside facility, or being referred to PCS for reasons other than their malignancy. Of the 183 patients referred to PCS who were eligible for inclusion, some (n = 65) did not attend their outpatient appointment and therefore never received PCS. Demographic, treatment, and outcome data were compared between these “no-PCS” patients to those of patients were able to attend their PCS appointment (n = 118). PCS treatment was defined as the attendance of at least one PCS appointment.

Demographic data on all patients was obtained through a prospectively-maintained institutional palliative care database. Additional data regarding marital status, ongoing oncologic treatments, ED visits, and hospitalizations was captured through a review of each patient’s electronic medical record (EMR, Epic Systems Corporation; Verona, Wisconsin). For the purpose of this analysis, treatment with chemotherapy referred to the active administration of systemic agents givenat any time during the treatment course. Treatment with radiation therapy was considered affirmative if treatment occurred at any time point throughout the patient’s treatment course. We defined a palliative procedure as any intervention performed following PCS referral with the intent of relieving symptoms and/or improving quality of life. Surgical procedures were those that were performed in an operating room, which necessitated an incision, and were staffed by a board eligible/board certified surgeon.

We assumed that terminal patients who had longer survival would have more opportunities to visit the ED and be admitted to hospital. To control for this confounder, the number of emergency visits, hospital admissions, and total length of stay was expressed as a rate per patient per 30 days of life. The number of ED visits, hospital visits and hospital days were analyzed via negative binomial regression. Regression models included age, sex, race, primary site, and PCS as fixed effects and log of follow up days as a model offset. Subgroup analyses were performed for colorectal cancer patients. Conditional risk ratios and 95% confidence intervals were reported for negative binomial regression models. It is possible that patients who did not receive PCS were unable to attend their appointments because of worse performance status.In order to control for potential treatment selection bias due to patient performance status, a sensitivity analysis was done excludingpatients (n = 25) who died within 30 days of PCS referral.

Survival duration was the time between date of initial PCS referral until date of death, date of last contact, or closing follow-up, if alive. The event for overall survival (OS) was death from any cause. In cases where the precise date of death was absent in the EMR, date of death was obtained from the institutional cancer registry.

Baseline comparisons between treatment groups were performed with t-tests and Fisher's exact tests for continuous and categorical variables, respectively. Patient survival was compared using Kaplan Meier and the log-rank test. Overall survival was also analyzed via multivariable Cox proportional hazards models. The Cox proportional hazards model included age, sex, race, primary site and PCS as fixed effects. Factors regarding cancer therapy were strongly correlated with primary site and weretherefore excluded from the multivariable models. Marital and insurance status were also removed from multivariable analyses to reduce multicollinearity, after confirming that there were no significant imbalances in these factors across treatment groups and that they were not significantly associated with outcomes. Outcomes for all GI malignancies were analyzed together, controlling for primary site. Additionally, we conducted a subgroup analysis of patients with colorectal cancer, which was the most common primary site in the study. Conditional hazard ratios and 95% confidence intervals were reported for proportional hazard models. Statistical significance was determined at the alpha = 0.05 level. All statistical analyses were completed in SAS version 9.4.

### Ethics approval

This study was performed in line with the principles of the Louisiana State University Health Science Center Institutional Review Board, and it was felt to be exempt research (Protocol No. 19-1396).

## Results

Between 2014 and 2018, 183 patients with terminal HPB or GI cancer were referred for PCS at our institution.Patient demographics are outlined in Table 1. The mean age of patients was 64 years. 101 (55.2%) patients were male, and 96 (52.5%) patients were white. The most prevalent cancer type was metastatic colorectal cancer (41%), followed by pancreatic cancer (19.1%), and liver or bile duct cancers (16.4%). Other cancer types included 34 patients (19%) with gastroesophageal malignancy and patients with advanced anal cancer (n = 6) or small bowel cancer (n = 3).

**Table 1:** Patient characteristics for patients with metastatic hepatopancreaticobiliary and gastrointestinal cancers referred to palliative care services at a single institution, 2014–2018.

	All Patients	PCS	No PCS	
	(n = 183)	(n = 118)	(n = 65)	*p*-value
Age (mean)	64.6 ± 12	64.1 ± 12.3	65.4 ± 12	0.5002
**Sex**				0.7266
Female	82 (44.8%)	54 (45.8%)	28 (43.1%)	
Male	101 (55.2%)	64 (54.2%)	37 (56.9%)	
**Race**				0.7228
White	96 (52.5%)	63 (53.4%)	33 (50.8%)	
Black	79 (43.2%)	49 (41.5%)	30 (46.2%)	
Other	8 (4.4%)	6 (5.1%)	2 (3.1%)	
**Married**				0.8342
Yes	92 (51.3%)	60 (50.9%)	32 (49.2%)	
No*	91 (49.7%)	58 (49.2%)	33 (50.8%)	
**Insurance**				0.9590
Yes	163 (89.1%)	105 (89%)	58 (89.2%)	
No*	20 (10.9%)	13 (11%)	7 (10.8%)	
**Site**				0.0082
Colorectal	75 (41%)	58 (49.2%)	17 (26.2%)	
Liver/Bile	30 (16.4%)	18 (15.3%)	12 (18.5%)	
Pancreas	35 (19.1%)	22 (18.6%)	13 (20%)	
Other	43 (23.5%)	20 (17%)	23 (35.4%)	
**Chemotherapy**				0.0098
Yes	136 (74.3%)	95 (80.5%)	41 (63.1%)	
No*	47 (25.7%)	23 (19.5%)	24 (36.9%)	
**Radiation**				0.3076
Yes	71 (38.8%)	49 (41.5%)	22 (33.9%)	
No*	112 (61.2%)	69 (58.5%)	43 (66.2%)	
**Palliative procedure**				0.6594
None*	105 (57.4%)	70 (59.3%)	35 (53.9%)	
Non-surgical	49 (27.6%)	29 (24.6%)	20 (30.8%)	
Surgical	29 (15.9%)	19 (16/1%)	10 (15.4%)	

*PCS* Palliative care services.

*Includes patients with unknown status.

Palliative procedures were performed in78 (42.6%) patients referred to PCS. In most cases (49/78, 62.8%) the procedure was non-surgical. These procedures included intestinal/biliary stents (n = 21), ureteral stents (n = 5), percutaneous nephrostomy tube (n = 1), peg/venting G tube (n = 9), tracheostomy (n = 1), IVC filter (n = 1), paracentesis/thoracentesis (n = 17). Palliative surgery was performed in 29 (15.9%) patients. The most common indication was a palliative colostomy for colorectal cancer (n = 25). There was no difference in gender, race, marital status, insurance coverage, or treatment type between patients who receivedpalliative procedure and those who did not. On average, palliative procedures were more often performed on men (51.5% vs. 31.7%, *p* = 0.0071) and on younger patients (60.4 vs. 67.7 years old, *p* < 0.0001).

Of the 183 patients referred to PCS, 118 (64.5%) patients attended their appointment and therefore received PCS. Comparison of patients who received PCS versus those who did not is summarized in Table 1. There was a higher percentage of patients with colorectal cancer amongst those who received PCS (49.2% vs. 26.2%, *p* = 0.0082) compared to those who did not receive PCS. Those who received PCS were more likely to be receiving chemotherapy at the time of their referral (80.5% vs. 63.1%, *p* = 0.0098). However, there were no significant differences in age, race, or gender. The proportion of patients receiving radiation therapy was also not significantly different. The proportion of patients that was uninsured was similar across treatment groups (11% vs. 10.8%, *p* = 0.9590), as was the percentage of patients who were married (50.9% vs. 49.2%, *p* = 0.8432).

Among all 183 patients referred to PCS, there were 294 total ED visits documented (1.6/patient) from the time of referral to last follow up. Similarly, there were 250 hospitalizations admissions (1.4/patient) documented at our institution during that time period. Theaverage number of hospitalsdays was 7per patient which represents a total of 1,256 inpatient days in aggregate. Because longer survival is associated with more opportunities for ED visits and hospitalizations, we reported this per patient per 30 days of life using a rate regression model offset to control for the number of life days (Table 2). The median number of ED visits/30 lifedays for all patients was 0.14. Controlling for age, sex, race, and primary site, the rate of ED visits during end-of-life period was significantlylower amongst patients who received PCS (RR = 0.62 (0.41–0.96); *p* = 0.0319). Similarly, the median number of hospital admissions/30 lifedays for all patients was 0.12, and controlling for age, sex, race, and primary site, the rate wassignificantly lower among patients who received PCS (RR = 0.44 (0.29–0.67), *p* = 0.0002). Finally, the median number of hospital days/30 lifedays was 0.48, and controlling for age, sex, race, and primary site, the rate wassignificantly lower amongst patients who received PCS (RR = 0.29 (0.16–0.53), *p* < 0.0001).

**Table 2:** Summary of life metrics and relative risk from negative binomial regression controlling for age, sex, race, and primary site for patients referred to palliative care services at a single institution, 2014–2018.

	All patients	PCS	No PCS	RR	95% CI	*p*-value
	Median (n = 181)	Median (n = 118)	Median (n = 63)			
ED Visits/30 life days	0.14	0.14	0.26	0.62	0.41–0.96	0.0319
Hospital Admissions/30 life days	0.12	0.09	0.34	0.44	0.29–0.67	0.0002
Inpatient Days/30 life days	0.48	0.36	1.41	0.29	0.16–0.53	< .0001

*PCS* Palliative care services, *RR* Risk ratio, *CI* Confidence interval, *ED* Emergency department.

**Figure 1:**
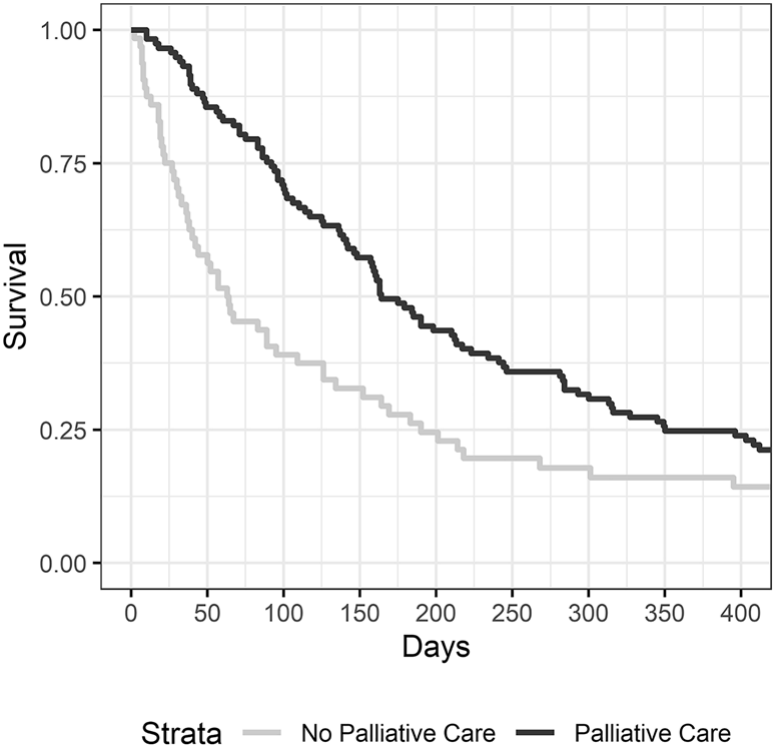
Kaplan Meier survival curves for patients with metastatic hepatopancreaticobiliary and gastrointestinal cancers referred to palliative care services at a single institution, 2014–2018.

**Figure 2:**
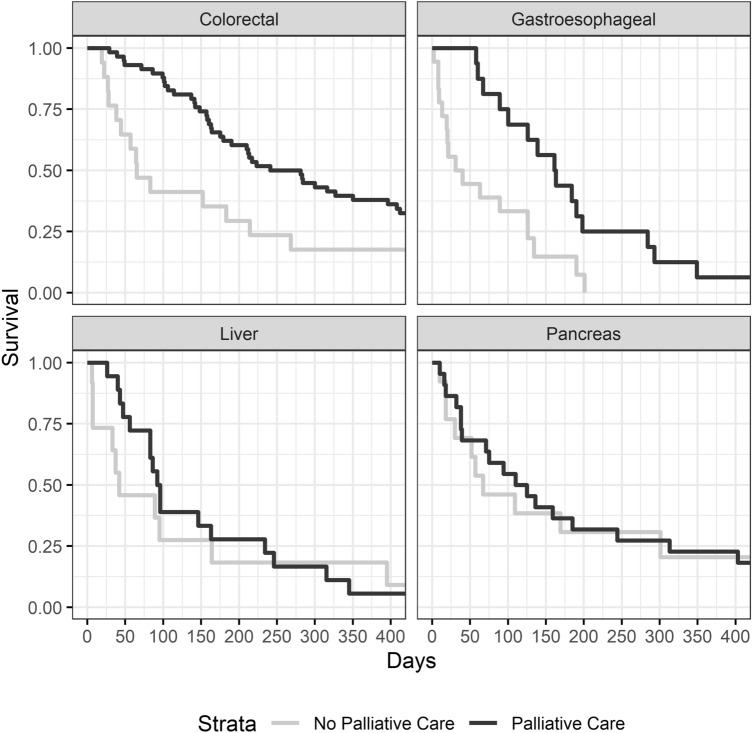
Kaplan Meier survival curves for patients with metastatic hepatopancreaticobiliary and gastrointestinal cancers referred to palliative care services at a single institution, 2014–2018, by primary tumor site.

The median follow-up of study patients was 139 days (range 2–1756). The majority of patients died within the study observation period (n = 162). The median overall survival of all patients referred to PCS was 142 days, and unadjusted overall survival was significantly longer among patients who received PCS versus those that did not (164 vs. 64 days, *p* = 0.0012, Fig. 1). Overall survival was further subset analyzed according to the primary tumor location (Fig. 2). When looking exclusively at patients with colorectal cancer, survival was significantly longer amongst patients receiving PCS (261 vs. 65 days, *p* = 0.0102). When looking exclusively at patients with gastroesophageal cancer, survival was also significantly longer amongst patients receiving PCS (162 vs. 35.5 days, *p* = 0.0037). However, when looking exclusively at patients with pancreatic cancer or liver/bile duct cancer, survival was not significantly affected by receipt of PCS (pancreatic cancer: *p* = 0.8810, liver/bile duct cancer: *p* = 0.7755).

Multivariate analysis of all patients referred to PCS was performed in order to identify risk factors for overall survival. These are outlined in Tables 3 and 4. PCS was associated with improved survival in the entire patient cohort (HR: 0.65 (0.46–0.92)), and when looking exclusively at colorectal cancer patients the protective effect of PCS was greater (HR: 0.43 (0.23–0.78)). In order to control for patients who may not have attended the PCS appointment because of an especially aggressive malignancy and/or poor performance status at the time of referral, multivariate analyses for survival was repeated including only patients who lived greater than 30 days from PCS referral. In each model, the association between PCS and improved overall survival trended in the same direction, though it was less in magnitude and no longer statistically significant [overall(n = 158):HR = 0.85 (0.57–1.27)], colorectal (n = 70):HR = 0.55 (0.28–1.09)].

**Table 3:** Multivariable model of survival among patients referred to palliative care services at a single institution, 2014–2018.

	All patients		Colorectal cancer patients	
	(n = 183)		(n = 75)	
	HR	95% CI	HR	95% CI
Age (1 year)	1.01	1.00–1.03	1.00	0.98–1.02
Male versus female	1.45	1.05–2.01	1.24	0.75–2.05
White versus other	0.83	0.60–1.13	1.71	1.00–2.92
Pancreatic versus colorectal	1.55	0.98–2.45	–	–
Liver versus colorectal	2.04	1.30–3.19	–	–
Other versus colorectal	1.76	1.15–2.70	–	–
PCS visit versus no PCS visit	0.65	0.46–0.92	0.43	0.23–0.78

*PCS* Palliative care services, *HR* Hazard ratio, *CI* Confidence interval.

**Table 4:** Multivariable model of survival among patients referred to palliative care services and survived at least 30 days after referral, at a single institution, 2014–2018.

	All patients		Colorectal cancer patients	
	(n = 158)		(n = 70)	
	HR	95% CI	HR	95% CI
Age (1 year)	1.01	1.00–1.03	1.00	0.98–1.02
Male visit female	1.32	0.92–1.89	1.25	0.74–2.10
White visit other	0.83	0.59–1.17	1.56	0.89–2.74
Pancreatic visit colorectal	1.51	0.92–2.48	–	–
Liver visit colorectal	2.16	1.34–3.49	–	–
Other visit colorectal	1.76	1.09–2.82	–	–
PCS visit visit no PCS visit	0.85	0.57–1.27	0.55	0.28–1.09

*PCS* Palliative care services, *HR* Hazard ratio, *CI* Confidence interval.

## Discussion

PCS in the United States has become a broad-based interdisciplinary medical specialty that addresses the needs of gravely ill patients and their families. Its goal is to optimize quality of life (QoL) by anticipating, preventing, and treating suffering through effective management of pain and other symptoms. PCS has seen rapid growth within the last two decades, and it is now considered an essential component to the cancer care continuum^15^.

While the positive influence of PCS on terminal cancer patients has been demonstrated by many investigators, most have focused on select cancer types or on large heterogenous groups of patients with varying types of cancer^16–21^. However, the impact of PCS on the subset of cancer patients with HPB and GI cancers has been less well studied^22–24^.In fact, at the time of this writing we have identified no other reports in the literature containing comparative data looking at the utility of PCS and its impact on outcomes and survival in HPB/UGI patients. It is possible that the benefit of PCS may be amplified in certain types of symptomatic cancers—like lung cancer or head and neck cancer—while adding very little to other types of malignancies such as pancreatic, hepatobiliary, and metastatic colon cancer.

This study was undertaken in an attempt to determine an association between PCS and end-of-life metrics in patients with terminal GI or HPB cancers. We focused our analysis on those referred to the outpatient PCS clinic over a 3.5-year period from 2014 to 2018. All study patients had incurable, advanced cancer and were referred to PCS for end-of-life care. We excluded those seen by PCS as inpatients as this group was assumed to be more moribund and therefore less likely to be impacted by PCS. Patients referred to PCS demonstrated an overall absentee rate of 35% that was relatively constant throughout the study period. We compared the outcomes of these absent (non-PCS) patients to those of a similar group who received PCS. A comparison of QoL metrics was not possible because scant data on this was available for the non-PCS patients. Instead, we used ED visits and hospital admissions as our primary outcomes analyzing these metrics as a function of 30 days of life.

Unnecessary ED visits overwhelm the health care system with a disproportionate consumption of finite resources. Additionally, structural barriers such as long wait times, competing demands, and a chaotic environment, undermine the provision of optimal palliative care in the ED^25^.

Overall, we found that patients receiving PCS required 38% fewer ED visits (Table 4); they needed 56% fewer hospital admissions; and they had 71% fewer total aggregate days in the hospital prior to death. We noted no significant association between receipt of PCS and age, gender, race, or marital status. Nor was receipt of PCS significantly impacted by insurance status.

It is possible that patients who did not receive PCS were unable to attend their appointments because of poor performance status. This was not possible to ascertain since no data on performance status existed on the non-PCS patients. We observed that non-PCS patients were less likely to be receiving chemotherapy at referral, and some investigators have shown that patients with poor performance status are less likely to consent to palliative chemotherapy^26^. Thus, one could infer that the reduced use of chemotherapy in the non-PCS group is indicative of worse performance status. However, to control for this possibility, we reanalyzed the groups excluding patients who survived less than 30 days from PCS referral. When this was done, PCS was still noted to be protective with fewer ER visits, fewer hospital visits, and fewer overall hospital admission days. Additionally, the use of palliative procedures in advanced cancer patients is directly correlated with improved performance status^27^. Thus, if a major difference in performance status existed in our study, then one would expect fewer palliative procedures among non-PCS patients, but this was not observed. Instead, 46.1% of non-PCS patients underwent a palliative procedure compared with 40.7% of patients receiving PCS.

Median survival was over three months longer among patients who received PCS (Fig. 1), and PCS was an independent predictor of improved survival on multivariate analysis. However, notable differences exist between the two comparison groups: the chief one is a higher percentage of patients with metastatic colorectal cancer amongst those who received PCS. It is common oncologic knowledge that patients with metastatic colorectal cancer as a group tend to have better survival than those with other types of metastatic HPB malignancies^28,29^. Therefore, it is possible that the difference in survival we observed is simply related to the distribution of a more indolent metastatic malignancy among patients who received PCS. However, in order to account for this possibility, we performed sensitivity analyses. Firstly, we compared survival of colorectal cancer patients to that of non-colorectal patients, confirming that those with non-colorectal cancer had worse survival than colorectal cancer patients. We then evaluated the association of PCS among only patients with metastatic colorectal cancer, and we found that PCS was still associated with improved overall survival, and it was still associated with fewer ED visits, hospital admissions, and total inpatient days. Subsequently, we excluded colorectal cancer patients and examined survival exclusively in non-colorectal patients, and we found that gastroesophageal cancer patients receiving PCS also experienced improved overall survival. However, when we looked at the subset of patients with pancreatic cancer or hepatobiliary cancer, while a trend towards significance was seen favoring PCS patients, no statistically significant survival benefit was appreciated. The reasons for this are not clear, and to date very little has been written on the effectiveness of PCS in pancreatic cancer patients^30^. It may be that the aggressive, systemic nature of these cancers is such that survival of these patients is simply unaffected by PCS. Another possibility is that PCS does impact survival in pancreatic or hepatobiliary cancer, but the difference was not detectable given the small power of our subset analysis. Regardless, we intend to investigate this in the future with a larger, more mature data set.

In summary, this is one of very few studies evaluating the role of PCS amongst patients with HPB/GI cancers. PCS was associated with fewer ER visits, fewer hospital admissions, and fewer total hospital days, along with prolonged overall survival. We feel that this data should encourage practitioners to refer patients with terminal HPB/GI cancer to PCS early in their end-of-life course.

## Data Availability

The authors will provide a deidentified dataset to the researchers upon request. Please contact Dr. John Lyons at john.lyons@fmolhs.org with any data requests.

## References

[1] Report on a National Survey Concerning Patients with Cancer Nursed at Home - PMC (accessed 3 June 2022); https://www.ncbi.nlm.nih.gov/pmc/articles/PMC2480023/

[2] Bean WB (1961). Peace at the last: A survey of terminal care in the United Kingdom; a report to the Calouste Gulbenkian Foundation 1960. Arch. Intern. Med..

[3] Hinton JM (1963). The physical and menial distress of the dying. QJM.

[4] Kubler-Ross E (1969). On Death and Dying.

[5] Connor SR (2007). Development of hospice and palliative care in the United States. Omega.

[6] Hughes MT, Smith TJ (2014). The growth of palliative care in the United States. Annu. Rev. Public Health.

[7] Haun MW (2017). Early palliative care for adults with advanced cancer. Cochrane Database Syst. Rev..

[8] Temel JS (2010). Early palliative care for patients with metastatic non–small-cell lung cancer. N. Engl. J. Med..

[9] Greer JA (2012). Effect of early palliative care on chemotherapy use and end-of-life care in patients with metastatic non-small-cell lung cancer. J. Clin. Oncol..

[10] Hoerger M (2018). Defining the elements of early palliative care that are associated with patient-reported outcomes and the delivery of end-of-life care. J. Clin. Oncol..

[11] Morrison RS (2011). Palliative care consultation teams cut hospital costs for medicaid beneficiaries. Health Aff. (Millwood).

[12] Ferrell BR (2017). Integration of palliative care into standard oncology care: American society of clinical oncology clinical practice guideline update. J. Clin. Oncol..

[13] Smart, S., Miller, J., Barry, R. & Lyons, J. Palliative care in the outpatient setting: focus on head and neck cancers. in *American Academy of Otolaryngology-Head and Neck Surgery Annual Meeting and OTO Expo* (2017).

[14] Cheatle MD, Compton PA, Dhingra L, Wasser TE, O’Brien CP (2019). Development of the revised opioid risk tool to predict opioid use disorder in patients with chronic nonmalignant pain. J. Pain.

[15] Dans M (2021). NCCN Guidelines® insights: Palliative care, version 2.2021. J. Natl. Compr. Canc. Netw..

[16] Sullivan DR (2019). Association of early palliative care use with survival and place of death among patients with advanced lung cancer receiving care in the veterans health administration. JAMA Oncol..

[17] Lee YJ (2015). Association between the duration of palliative care service and survival in terminal cancer patients. Support. Care Cancer.

[18] Bakitas M (2009). Effects of a palliative care intervention on clinical outcomes in patients with advanced cancer: the Project ENABLE II randomized controlled trial. JAMA.

[19] Rugno FC, Paiva BSR, Paiva CE (2014). Early integration of palliative care facilitates the discontinuation of anticancer treatment in women with advanced breast or gynecologic cancers. Gynecol. Oncol..

[20] Lowery WJ (2013). Cost-effectiveness of early palliative care intervention in recurrent platinum-resistant ovarian cancer. Gynecol. Oncol..

[21] Mulvey CL, Smith TJ, Gourin CG (2016). Use of inpatient palliative care services in patients with metastatic incurable head and neck cancer. Head Neck.

[22] Costi R, Leonardi F, Zanoni D, Violi V, Roncoroni L (2014). Palliative care and end-stage colorectal cancer management: the surgeon meets the oncologist. World J. Gastroenterol..

[23] Perone JA, Riall TS, Olino K (2016). Palliative care for pancreatic and periampullary cancer. Surg. Clin. N. Am..

[24] Woodrell CD, Hansen L, Schiano TD, Goldstein NE (2018). Palliative care for people with hepatocellular carcinoma, and specific benefits for older adults. Clin. Ther..

[25] Lamba S, Quest TE (2011). Hospice care and the emergency department: rules, regulations, and referrals. Ann. Emerg. Med..

[26] Murakawa Y, Sakayori M, Otsuka K (2019). Impact of palliative chemotherapy and best supportive care on overall survival and length of hospitalization in patients with incurable Cancer: a 4-year single institution experience in Japan. BMC Palliat. Care.

[27] Miner TJ, Brennan MF, Jaques DP, Wood WC (2004). A prospective, symptom related, outcomes analysis of 1022 palliative procedures for advanced cancer. Ann. Surg..

[28] Weiser MR (2018). AJCC 8th edition: Colorectal cancer. Ann. Surg. Oncol..

[29] Kwon W (2018). Multinational validation of the American Joint Committee on 8th edition cancer pancreatic cancer staging system in a pancreas head cancer cohort. J. Hepatobiliary Pancreat. Sci..

[30] Sheffield KM (2011). End-of-life care in medicare beneficiaries dying with pancreatic cancer. Cancer.

